# Mesh fixation to fascia during incisional hernia repair results in increased prevalence of pain at long-term follow up: a multicenter propensity score matched prospective observational study

**DOI:** 10.1007/s00464-021-08355-8

**Published:** 2021-02-23

**Authors:** Andreas Kohler, Joël L. Lavanchy, Rahel Gasser, Roland Wyss, Lars Nowak, Andreas Scheiwiller, Peter Hämmerli, Daniel Candinas, Guido Beldi

**Affiliations:** 1 Department of Visceral Surgery and Medicine, Inselspital, Bern University Hospital, University of Bern, Bern, Switzerland; 2 Department of Visceral and Thoracic Surgery, Kantonsspital Winterthur, Winterthur, Switzerland; 3 Department of Surgery, Spital Grabs, Grabs, Switzerland; 4 Department of General and Visceral Surgery, Kantonsspital Luzern, Luzern, Switzerland; 5 Department of Surgery, Spital Walenstadt, Walenstadt, Switzerland

**Keywords:** Incisional hernia, Mesh fixation, Pain, Long-term follow up, Multicenter study, Propensity score matching

## Abstract

**Background:**

Patient-reported outcomes such as postoperative pain are critical for the evaluation of outcomes after incisional hernia repair. The aim of this study is to determine the long-term impact of mesh fixation on postoperative pain in patients operated by open and laparoscopic technique.

**Methods:**

A multicenter prospective observational cohort study was conducted from September 2011 until March 2016 in nine hospitals across Switzerland. Patients undergoing elective incisional hernia repair were included in this study and stratified by either laparoscopic or open surgical technique. Propensity score matching was applied to balance the differences in baseline characteristics between the treatment groups. Clinical follow-up was conducted 3, 12 and 36 months postoperatively to detect hernia recurrence, postoperative pain and complications.

**Results:**

Three-hundred-sixty-one patients were included into the study. No significant differences in hernia recurrence and pain at 3, 12 and 36 months postoperatively were observed when comparing the laparoscopic with the open treatment group. Mesh fixation by sutures to fascia versus other mesh fixation led to significantly more pain at 36 months postoperatively (32.8% vs 15.7%, *p* = 0.025).

**Conclusions:**

At long-term follow-up, no difference in pain was identified between open and laparoscopic incisional hernia repair. Mesh fixation by sutures to fascia was identified to be associated with increased pain 36 months after surgery. Omitting mesh fixation by sutures to the fascia may reduce long-term postoperative pain after hernia repair.

With an incidence of up to 25%, incisional hernia is a frequent long-term complication of open abdominal surgery [1, 2]. Treatment of incisional hernia and related complications induces considerable health-care spending [3, 4].

International guidelines equivocally advocate for an incisional hernia repair with a non-absorbable mesh, because recurrence is two-fold reduced compared to suture repair [5, 6]. Laparoscopic operation technique was shown to reduce postoperative surgical site infections (SSI), overall complications and hospital length of stay. However, recurrence rates do not differ when comparing open and laparoscopic operation technique [7–9].

Long-term patient-reported outcomes remain to be studied in detail to refine surgical techniques [10]. With up to 50% of patients that underwent incisional hernia repair reporting pain at 6 months follow-up this remains a substantial problem [11, 12]. Further, the ideal mesh position (onlay vs. sublay vs. intraperitoneal) and fixation technique has broadly been discussed in the literature with regard to recurrence of incisional hernia [13–15]. However, these studies often neglect patient-reported outcomes.

We hypothesized that type of mesh fixation technique using tacks, suture fixation to the fascia or the peritoneum is of major importance for the development of chronic pain and might be more important than the actual mesh position.

Therefore, we aimed to report long-term outcomes of the applied surgical techniques, mesh position and fixation technique and assess their relationship with the occurrence of long-term pain.

## Material and methods

### Study design

A prospective observational study including patients that underwent open or laparoscopic incisional hernia repair was conducted in nine hospitals across Switzerland. Patients were included from September 2010 to March 2016, inclusion criteria were age ≥ 18 years and written informed consent. Exclusion criteria comprised emergency surgery and local or systemic infection at the time of surgery. The primary outcome was recurrence at 3 years after surgery. Secondary endpoints included pain scores on a visual analogue scale (VAS, range 0–10), localization of pain, consumption of pain killers, SSI graded according to the definition of the Center for Disease Control [16] and overall complication graded according to Dindo-Clavien [17].

### Data collection

Following parameters were meticulously recorded during surgery: surgical access (laparoscopic vs. open), mesh material (Polypropylene vs. Polyester vs. other), mesh position (onlay vs. sublay vs. intraperitoneal) and mesh fixation (suture to the facia, suture to the peritoneum and tacks).

Follow-up data were collected during patient visits in the outpatient clinic 3, 12 and 36 months after surgery. Patients completed a standardized questionnaire and a clinical examination of the abdominal wall was performed. If there was a doubt regarding hernia recurrence, imaging studies were ordered.

### Statistical analysis

Categorical variables are reported as numbers and percentages, continuous variables as median and interquartile range (IQR). Statistical differences were analyzed using Fisher’s exact test and Mann–Whitney-*U* test, respectively. A *p*-value ≤ 0.05 was considered statistically significant. Propensity score matching was performed by matching for age, sex, body-mass-index (BMI), American Society of Anesthesiologists (ASA) score, hernia size and site of primary incision. Propensity score matching was applied using the *MatchIt* package for *R* [18] and Euler diagrams were plotted using the *eulerr* package for *R* [19]. All other statistical analyses were performed using SPSS Version 25 (IBM, Armonk, NY).

## Results

**Fig. 1:**
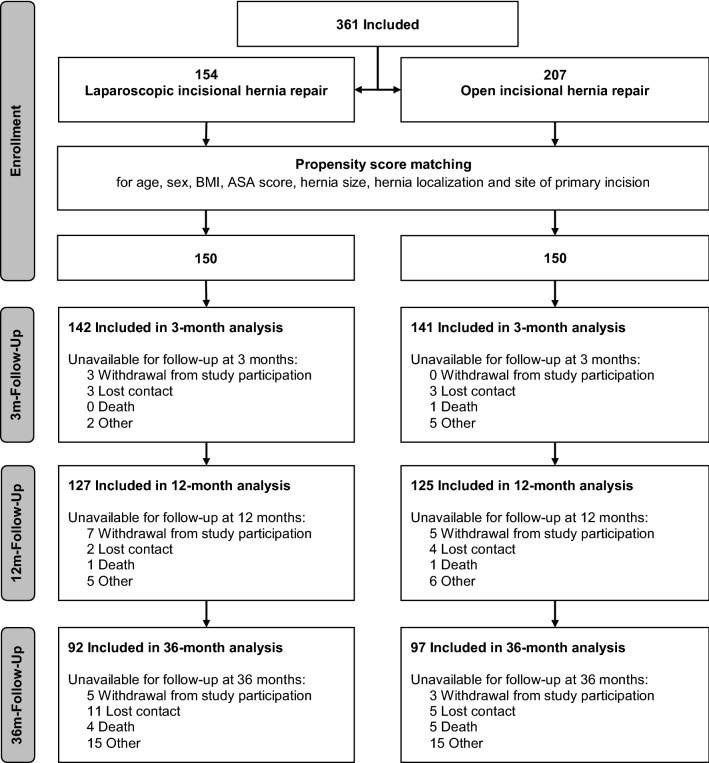
Patient flow-chart

During the 66 months study period, 361 patients with a median age of 64.0 (IQR 55.0–71.0) years and a median BMI of 28.7 (25.4–33.0) kg/m^2^ were enrolled. Thereof, 154 (42.7%) patients underwent laparoscopic and 207 (57.3%) patients open incisional hernia repair. The detailed flow-chart of the study is displayed in Fig. 1. Baseline characteristics of the study groups differed significantly in hernia size and site of primary incision. After propensity score matching no significant differences between the laparoscopic and the open group were left (Table 1).

**Table 1:** Baseline characteristics of the study population

	Crude			Matched^c^		
	Lap. (*n* = 154)	Open (*n* = 207)	*p*-value	Lap. (*n* = 150)	Open (*n* = 150)	*p*-value
Age, years, median (IQR)	64 (54–70)	64 (57–71)	0.434^a^	64 (54–70)	65 (57–71)	0.359^a^
Sex, female (%)/male (%)	68 (44.2)/86 (55.8)	83 (39.4)/124 (60.1)	0.452^b^	66 (44.0)/84 (56.0)	64 (42.7)/86 (57.3)	0.907^b^
BMI, kg/m^2^, median (IQR)	29.2 (25.2–33.0)	28.4 (25.3–33.0)	0.856^a^	29.1 (25.2–33.1)	28.4 (24.7–32.3)	0.694^a^
ASA score, *n* (%)			0.775^b^			0.915^b^
1	12 (7.8)	16 (8.5)		12 (8.0)	12 (8.0)	
2	95 (61.7)	112 (59.6)		94 (62.7)	91 (60.7)	
3	42 (27.3)	56 (29.8)		42 (28.0)	46 (30.7)	
4	2 (1.3)	1 (0.5)		2 (1.3)	1 (0.7)	
Missing	3 (1.9)	3 (1.6)		–	–	
Hernia size, cran.-caud., cm, median (IQR)	3.0 (2.0–6.0)	5.0 (3.0–10.5)	< 0.001^a^	3.0 (2.0–6.0)	5.0 (2.0–7.0)	0.068^a^
Hernia localization, *n* (%)			0.220^b^			1.00^b^
Median	123 (79.9)	163 (78.7)		124 (82.7)	123 (82.0)	
Lateral	26 (16.9)	42 (20.3)		26 (17.3)	27 (18.0)	
Missing	5 (3.2)	2 (1.0)		–	–	
Site of primary incision, *n* (%)			0.018^b^			0.307^b^
Median laparotomy	71 (46.1)	130 (62.8)		70 (46.7)	85 (56.7)	
Transverse laparotomy	28 (18.2)	26 (12.6)		28 (18.7)	20 (13.3)	
Laparoscopic access	33 (21.4)	26 (12.6)		32 (21.3)	25 (16.7)	
Other	21 (13.6)	24 (11.6)		20 (13.3)	20 (13.3)	
Missing	1 (0.6)	1 (0.5)		–	–	
Smoking, *n* (%)	46 (30.1)	47 (22.7)	0.144^b^	44 (29.3)	34 (22.7)	0.236^b^
Previous incisional hernia, *n* (%)	29 (19.0)	36 (17.4)	0.782^b^	28 (18.7)	30 (20.0)	0.884^b^

^a^Mann-Whitney-*U* test

^b^Fisher’s exact test

^c^Matched for age, sex, BMI, ASA score, hernia size and localization and primary incision

**Fig. 2:**
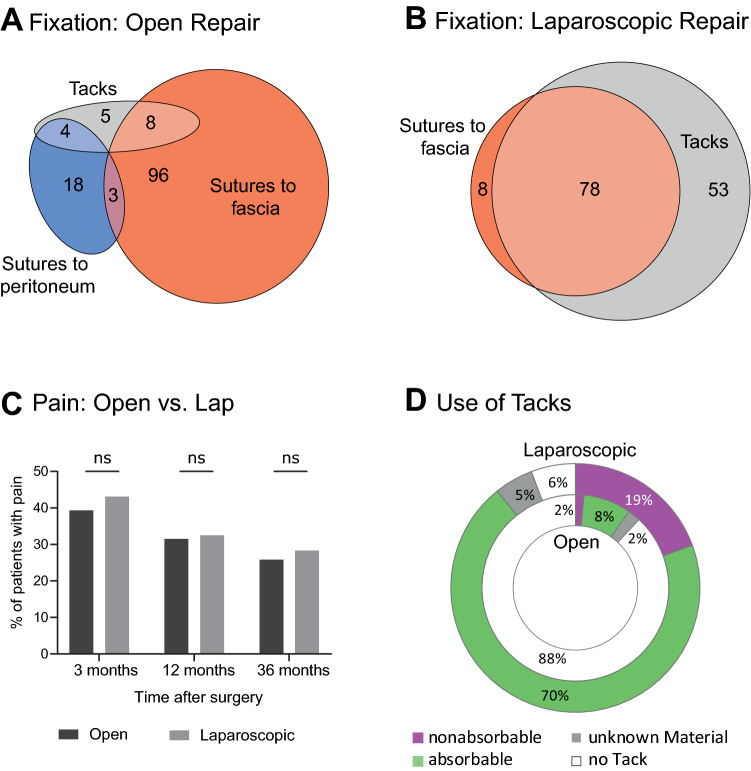
Open versus laparoscopic repair. **A**, **B** Mesh fixation techniques in open and laparoscopic incisional hernia repair. **C** Pain perception after open and laparoscopic repair up to 3 years. **D** Use of absorbable and nonabsorbable tacks in the two groups

The operative technique differed significantly between the two study groups (Table 2). In the laparoscopic group more polyester meshes were used (28.8% vs. 12.0%, *p* < 0.001), the mesh position was more frequently intraperitoneal (98.0% vs. 26.7%, *p* < 0.001) and tacks were more often used to fix the mesh (87.3% vs. 11.3%, *p* < 0.001) compared to the open group. Operation time was significantly shorter in the laparoscopic group (106 vs. 140 min, *p* < 0.001). Figure 2 shows the mesh fixation techniques for the two groups. While mesh fixation by sutures to the fascia was predominantly used in open operated cases, a combination of sutures to the fascia and tack fixation was used in most laparoscopic procedures.

**Table 2:** Operative technique

	Lap	Open	*p*-value
Type of mesh, *n* (%)			< 0.001^b^
Polypropylene	106 (70.7)	126 (84.0)	
Polyester	43 (28.7)	18 (12.0)	
Other	1 (0.7)	2 (1.3)	
No mesh	–	4 (2.7)	
Mesh position, *n* (%)			< 0.001^b^
Intraperitoneal	147 (98.0)	40 (26.7)	
Sublay	3 (2.0)	100 (66.7)	
Onlay	–	5 (3.3)	
Missing data	–	5 (3.3)	
Mesh fixation, *n* (%)			
Suture to fascia	86 (57.3)	107 (71.3)	0.003^b^
Suture to peritoneum	–	25 (16.7)	< 0.001^b^
Tacks	131 (87.3)	17 (11.3)	< 0.001^b^
Missing data	11 (7.3)	16 (10.6)	
Mesh size, cm^2^, median (IQR)	312 (225–600)	413 (225–600)	0.999^a^
Duration of the operation, min. median (IQR)	106 (70–135)	140 (92–193)	< 0.001^a^

^a^Mann-Whitney-*U* test

^b^Fisher’s exact test

During hospital stay, overall postoperative complications (9.3% vs. 21.3%, *p* = 0.006) and superficial SSI (0.7% vs. 5.3%, *p* = 0.036) were less frequent in the laparoscopic group compared to the open group (Table 3). Patients who underwent laparoscopic incisional hernia repair showed reduced median hospital length of stay (4 vs. 5 days, *p* < 0.001) compared to patients that underwent open incisional hernia repair.

**Table 3:** Outcomes at hospital discharge

	Lap	Open	*p*-value
Postoperative complications, *n* (%)	14 (9.3)	32 (21.3)	0.006^b^
Dindo-Clavien, *n* (%)			0.009^b^
Grade I	3 (2.0)	8 (5.3)	
Grade II	2 (1.3)	15 (10)	
Grade IIIa	1 (0.7)	2 (1.3)	
Grade IIIb	5 (3.3)	4 (2.7)	
Grade IVa	3 (2.0)	3 (2.0)	
Surgical site infections, *n* (%)			
Superficial	1 (0.7)	8 (5.3)	0.036^b^
Deep	–	1 (0.7)	1.000^b^
Organ/space	3 (2.0)	–	0.247^b^
Fistula formation, *n* (%)	1 (0.7)	–	1.000^b^
Seroma formation, *n* (%)	1 (0.7)	4 (2.7)	0.371^b^
Pain level, VAS, median (IQR)	2 (1–3)	2 (1–3)	0.104^a^
HLOS, days, median (IQR)	4 (2–5)	5 (3–8)	< 0.001^a^
Uptake normal daily activities, days, median (IQR)	21 (14–30)	24 (14–42)	0.063^a^

*VAS* Visual Analogue Scale, *HLOS* hospital length of stay

^a^Mann-Whitney-*U* test

^b^Fisher’s exact test

During the whole study period, there were no significant differences in hernia recurrence between laparoscopic and open incisional hernia repair. Frequency of hernia recurrence was not influenced by mesh fixation technique. Superficial SSI were less frequent in the laparoscopic group compared to the open group at three months postoperatively (4.4% vs. 14.8%, *p* = 0.004).

The prevalence of patients experiencing pain was similar between the open and laparoscopic group at 3, 12 and 36 months after surgery. In those patients experiencing pain, median pain level was significantly higher (3.5 vs. 2.0 VAS, *p* = 0.045) 12 months postoperatively in patients who underwent laparoscopic hernia repair. A similar trend was observed at 36 months after surgery.

**Fig. 3:**
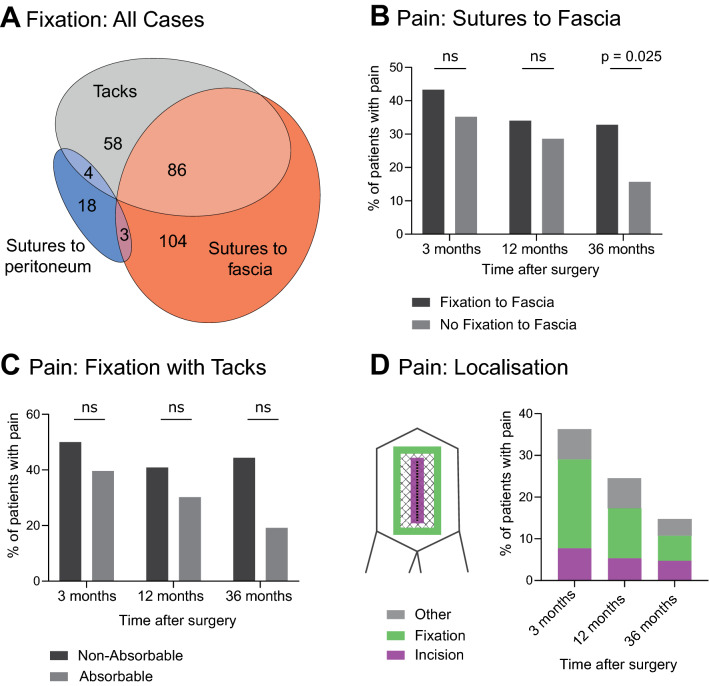
Pain levels according to fixation technique. **A** Mesh fixation for all cases. **B** Pain with vs. without mesh fixation by sutures to fascia. **C** Pain after mesh fixation by absorbable vs. non-absorbable tacks. **D** Site of pain localization after 3, 12 and 36 months

During the whole study period, the mesh position had no significant impact on the frequency of pain. However, the mesh fixation technique was of major importance. At 36 months after surgery, significantly more patients with mesh fixation by sutures to the fascia were in pain compared to patients without suture fixation to the fascia (32.8% vs 15.7%, *p* = 0.025), regardless of other fixation techniques used. Patients with absorbable tack fixation showed a trend toward less pain when compared with patients with non-absorbable tack fixation during the entire follow-up period (Fig. 3C). Detailed results of pain perception in the long-term follow-up are shown in Fig. 3 and Table 4.

**Table 4:** Pain perception and long-term complications

	Lap	Open	*p*-value
Preoperative			
Pain, *n* (%)	90 (62.1)	98 (66.2)	0.468^b^
Pain level, VAS, median (IQR)	4 (2–6)	3 (2–5)	0.089^a^
Pain killer consumption, *n* (%)	29 (19.3)	33 (22.0)	0.393^b^
Follow-up at 3 months			
Pain, *n* (%)	59 (43.1)	53 (39.3)	0.540^b^
Pain level, VAS, median (IQR)	3.0 (2.0–5.0)	3.0 (1.0–4.0)	0.138^a^
Pain killer consumption, *n* (%)	15 (10.6)	12 (8.5)	0.860^b^
Pain localization, *n* (%)			0.067^b^
Site of incision	6 (4.0)	17 (11.3)	
Site of fixation	39 (26.0)	25 (16.7)	
Other	12 (8.0)	10 (6.7)	
Surgical site infections, *n* (%)			
Superficial	6 (4.4)	20 (14.8)	0.004^b^
Deep	2 (1.5)	3 (2.2)	0.683^b^
Organ/space	1 (0.7)	–	1.000^b^
Fistula formation, *n* (%)	1 (0.7)	–	1.000^b^
Seroma formation, *n* (%)	10 (7.3)	15 (11.1)	0.301^b^
Bulging, *n* (%)	20 (14.6)	12 (8.9)	0.188^b^
Recurrence, *n* (%)	7 (5.1)	1 (0.7)	0.066^b^
Follow-up at 12 months			
Pain, *n* (%)	40 (32.5)	39 (31.5)	0.892^b^
Pain level, VAS, median (IQR)	3.5 (2.0–6.0)	2.0 (1.0–4.0)	0.045^a^
Pain killer consumption, *n* (%)	14 (11.0)	9 (7.2)	0.569^b^
Pain localization, *n* (%)			0.999^b^
Site of incision	8 (6.3)	8 (6.4)	
Site of fixation	18 (14.2)	18 (14.4)	
Other	10 (7.9)	11 (8.8)	
Fistula formation, *n* (%)	–	2 (1.6)	0.498^b^
Seroma formation, *n* (%)	6 (4.8)	15 (12.1)	0.066^b^
Recurrence, *n* (%)	15 (11.8)	8 (6.4)	0.188^b^
Follow-up at 36 months			
Pain, *n* (%)	26 (28.3)	23 (25.8)	0.740^b^
Pain level, VAS, median (IQR)	3.0 (2.0–4.0)	2.0 (1.8–4.0)	0.551^a^
Pain killer consumption, *n* (%)	8 (8.7)	4 (4.1)	0.544^b^
Pain localization, *n* (%)			0.674^b^
Site of incision	5 (5.4)	9 (9.3)	
Site of fixation	11 (12.0)	7 (7.2)	
Other	7 (7.6)	5 (5.2)	
Fistula formation, *n* (%)	–	1 (1.1)	0.497^b^
Seroma formation, *n* (%)	2 (2.2)	5 (5.5)	0.278^b^
Bulging, *n* (%)	23 (25.0)	15 (16.9)	0.204^b^
Recurrence, *n* (%)	9 (9.8)	14 (14.4)	0.686^b^

*VAS* Visual Analogue Scale

^a^Mann-Whitney-*U* test

^b^Fisher’s exact test

## Discussion

This propensity score matched prospective observational study was designed to comprehensively assess the outcome after open and laparoscopic incisional hernia repair. In addition to the surgical access, we investigated mesh position and fixation technique as factors for long-term postoperative pain.

The study confirms the well-known benefits of laparoscopic compared to open incisional hernia repair as reduced hospital length of stay, less complications and SSI at equal recurrence rates 3 years postoperatively [7]. The prevalence of pain up to 36 months after surgery was not different between the groups. The study demonstrates the advantages of peritoneal suture and tack fixation compared to sutures to the fascia regarding chronic postoperative pain.

Recently introduced endoscopic sublay techniques such as EMILOS [20] and eTEP [21] do not require mesh fixation by sutures or tacks and might therefore combine lower long-term pain levels with known advantages of laparoscopic hernia repair as fewer surgical site infections and shorter length of stay.

A Dutch randomized trial comparing three fixation techniques (absorbable sutures and tacks vs. tacks alone vs. non-absorbable sutures and tacks) in laparoscopic incisional and ventral hernia repair showed no differences in postoperative pain and quality of life [22]. However, this trial had a very limited follow-up of 3 months only. Similarly, we only found a trend for more pain 3 months after surgery; however, the difference became more evident during the long-term follow-up. An Indian randomized trial comparing transfascial suture versus tack and suture mesh fixation in laparoscopic incisional and ventral hernia repair revealed significantly lower pain levels in the suture group 1-month postoperatively [23]. However, clinical relevance of − 0.8 points VAS is questionable and no follow-up beyond 3 months is provided in this trial.

An international multicenter randomized trial comparing tacks alone versus transfascial suture and tack mesh fixation in laparoscopic ventral hernia repair showed less abdominal wall pain in the tacks alone group 3-month postoperatively [24]. Again, no follow-up regarding pain is provided beyond 3-month after surgery. Our study confirms the results of this trial. The absence of mesh fixation to the fascia reduces the postoperative pain even in the long-term after 36 months postoperatively.

A registry-based study from Germany found that pain at one-year follow-up after incisional hernia repair is associated with female sex [25]. In our study females had significantly more often pain at 3 months postoperatively compared to men (52.2% vs. 33.3%, *p* = 0.003). However, at 36 months postoperatively the frequency of pain was equal for both sexes.

A recent study from the Netherlands suggests that a structured interview on the phone is sensitive to detect recurrence after incisional hernia repair [26]. However, if the screening questions were answered positively patients were invited for a clinical follow-up in the cited study. A strength of the current study is the entire clinical follow-up, even though it might have reduced the follow-up rate.

The surgical technique differed considerably between the study groups. However, as this study aims to elaborate pain levels in relation to the type of mesh fixation, different operative techniques were permitted.

A limitation of the study is the high dropout rate. Whereas data of close to 85% of included patients is complete at 12 months follow-up, only 61% and 65% of patients presented for the 36 moths follow-up appointment. The study protocol requested a clinical follow-up 36 months after surgery, which might have been the reason for withdrawal from study participation for many participants in this population with 25% of patients above the age of 70. Further, over the 3-year period, dropout due to death and loss of contact were responsible for missing data at 36 months of follow-up.

Although propensity score matching is a valid statistical method, this study is limited due to its non-randomized design. Furthermore, the external validity of this study would be increased with a larger patient population.

## Conclusion

This propensity score matched prospective study did not show differences in the prevalence of pain at 3, 12 and 36 months after laparoscopic versus open incisional hernia repair. Mesh fixation by sutures to fascia was identified to be associated with increased pain 36 months after surgery. Therefore, mesh fixation by sutures to the fascia should be omitted to avoid long-term postoperative pain after hernia repair.
